# Enhanced resistance to heat and fungal infection in transgenic *Trichoderma* via over-expressing the HSP70 gene

**DOI:** 10.1186/s13568-024-01693-5

**Published:** 2024-04-10

**Authors:** Yanhua Huang, Changfa Liu, Xuexue Huo, Xianzhi Lai, Wentao Zhu, Yongren Hao, Zehui Zheng, Kai Guo

**Affiliations:** Biology Institute, Qilu University of Technology (Shandong Academy of Sciences), Jinan, 250014 China

**Keywords:** *Trichoderma viride*, TvHSP70, Heat stress, Anti-oxidant defence system, Anti-fungal activity

## Abstract

Heat stress is one of the major abiotic stresses affecting the growth, sporulation, colonization and survival of *Trichoderma viride*. This study aimed to gain a better insight into the underlying mechanism governing the heat stress response of *T*. *viride* Tv-1511. We analysed the transcriptomic changes of Tv-1511 under normal and heat stress conditions using RNA sequencing. We observed that Tv-1511 regulates the biosynthesis of secondary metabolites through a complex network of signalling pathways. Additionally, it significantly activates the anti-oxidant defence system, heat shock proteins and stress-response-related transcription factors in response to heat stress. *TvHSP70* was identified as a key gene, and transgenic Tv-1511 overexpressing *TvHSP70* (TvHSP70-OE) was generated. We conducted an integrated morphological, physiological and molecular analyses of the TvHSP70-OE and wild-type strains. We observed that *TvHSP70* over-expression significantly triggered the growth, anti-oxidant capacity, anti-fungal activity and growth-promoting ability of Tv-1511. Regarding anti-oxidant capacity, *TvHSP70* primarily up-regulated genes involved in enzymatic and non-enzymatic anti-oxidant systems. In terms of anti-fungal activity, *TvHSP70* primarily activated genes involved in the synthesis of enediyne, anti-fungal and aminoglycoside antibiotics. This study provides a comparative analysis of the functional significance and molecular mechanisms of HSP70 in *Trichoderma*. These findings provide a valuable foundation for further analyses.

**Supplementary Information:**

The online version contains supplementary material available at 10.1186/s13568-024-01693-5.

## Key points

Transcriptome analyses reveal the heat resistance mechanism of *Trichoderma*.

*HSP70* overexpression enhances growth-promoting ability and anti-oxidant capacities.

*HSP70* promotes anti-fungal resistance by up-regulating antibiotic synthesis genes.

**Supplementary Information:**

The online version contains supplementary material available at 10.1186/s13568-024-01693-5.

## Introduction

*Trichoderma*, a well-known filamentous fungi genus in agroecology, is ubiquitously present in soil and root ecosystems. They contain diverse secondary metabolites with various biological activities, and have extensive applications in industrial and agricultural production, as well as in environmental protection (Zhang et al. 2021; Abdelmoaty et al. 2021). *Trichoderma viride* has been isolated and selected as a biological control agent, widely recognised for its effectiveness in combating various plant pathogens, including cotton *Rhizoctonia solani* Kuhn (Gajera et al. 2020), *Macrophomina phaseolina* (Khan et al. 2021), *Macrophoma theicola branch canker* disease (Mareeswaran 2022), crown rot and powdery mildew diseases (Bhardwaj et al. 2022). Moreover, *T*. *viride* has also been used as an effective larvicide against dengue mosquitoes (Perera et al. 2023) and root-knot nematodes (Patil et al. 2021). In addition to its biocontrol potential in plants, *T*. *viride* also plays crucial roles in enhancing plant growth (Guo et al. 2020a), yield (Guo et al. 2020b) and stress tolerance (Datta et al. 2023; Li et al. 2022). Additionally, *T*. *viride* serves as the main enzyme-producing strain in the industry and is known to secrete large quantities of biomass-degrading enzymes (Li et al. 2023; Johnnie et al. 2021).

However, *Trichoderma* constantly face threats from various abiotic stresses in industrial and agricultural applications. Heat stress is one of the major abiotic stresses that harm *Trichoderma*. An increase in temperature causes a series of morphological, physiological, biochemical and molecular changes that adversely affect their growth, sporulation, colonisation and survival (Carro-Huerga et al. 2021), thereby limiting their large-scale application. Enhancing *Trichoderma*’s survival rate and efficacy under high-temperature conditions has become a crucial goal in the current climate change era. *Trichoderma* have developed various adaptation mechanisms, including heat avoidance, tolerance, recovery and escape, to survive in extreme temperature conditions (Poosapati et al. 2021). Recent evidence indicates that heat shock proteins (HSPs) are central to these heat tolerance mechanisms (Sarkar et al. 2021).

HSPs are highly conserved multi-functional molecules found in diverse organisms, ranging from prokaryotes to eukaryotes. Typically, they occur as stress-inducible isoforms rapidly synthesized in cells exposed to heat stress. Most living organisms respond to heat stress by regulating gene expression during transcription and/or translation processes to activate HSP synthesis (Feng et al. 2021). Primarily, HSPs act as binding partners to prevent protein denaturation or facilitate correct protein folding through folding/unfolding steps, thereby ensuring correct functional configuration (Luengo et al. 2019). Due to their high sensitivity to even minor stresses, HSPs are considered an early warning bio-indicators of cellular hazards (Harada et al. 2014). HSPs have been classified into six major classes based on their molecular weight: HSP100, HSP90, HSP70, HSP60, HSP40 and small heat shock proteins (sHSPs). Among these, HSP70 is the most studied isoform and has been implicated in diverse aspects of cellular protein homeostasis and adaptive immune responses (Zhou et al. 2023). HSP70 is a highly conserved protein among various species. Structurally, HSP70 comprises a 44 kDa N-terminal ATPase domain and a 25 kDa C-terminal peptide-binding domain (Harada et al. 2014), and its expression can be constitutive and/or inducible, depending on the conditions. Under normal conditions, HSP70 is constitutively expressed in cells and acts as an important molecular chaperone. However, under stress conditions, HSP70 expression is strongly induced in response to these stressors (Zhou et al. 2023). Moreover, HSP70 exhibits different functions based on its cellular location. Intracellular HSP70 plays cytoprotective roles as a chaperone protein, whereas extracellular HSP70 exerts immune-modulatory activity via paired receptors Siglec-5 and Siglec-14 (Jerry et al. 2015).

The functions of HSP70 in immune responses have been extensively explored at the molecular level. Previous studies have proposed over-expressing HSP70 as an effective method to induce thermal tolerance and improve resistance to adverse conditions (D’Souza et al. 2022; Ni et al. 2021). However, few studies have focused on the effects of HSP70 on *Trichoderma*. Recent advancements in transcriptome analysis, which integrates multiple types of morphological, physiological and molecular analyses, has been rapidly widely accepted as a powerful tool for uncovering the underlying mechanism of action (Huang et al. 2022). Therefore, we conducted an RNA-sequencing analysis to compare the transcriptomes of *T*. *viride* Tv-1511 under normal and heat stress conditions to elucidate the mechanism of heat resistance. Subsequently, we identified *TvHSP70* as a key gene involved in the heat shock response of *T*. *viride* Tv-1511. Finally, we detected the morphological and physiological changes resulting from the over-expression of *HSP70* gene, providing insights into the molecular mechanisms governing the functions of *HSP70* in *viride’*s growth, heat stress tolerance and anti-fungal activity.

## Materials and methods

### Fungal strains and plant materials

The *T*. *viride* strain Tv-1511 was screened from tobacco rhizosphere soil samples in our laboratory and registered with the China General Microbial Collection Center (Beijing, China) under the preservation number CGMCC No. 16,800. Matured spores were collected and stored at − 80℃. Cucumbers (*Cucumis sativus* L. cv. Jinyan No. 4) were grown in a 25 °C artificial climate chest under a 16/8-h light/dark cycle.

### Heat stress treatment and transcriptomic analysis

The mycelium of *T*. *viride* Tv-1511 underwent a heat stress test. In the constant heat stress assay, Tv-1511 mycelium was inoculated on potato dextrose agar (PDA) plates or into potato dextrose (PD) liquid medium and cultured at constant temperatures of 28℃, 33℃, 35℃ and 37℃. In the short-term heat shock experiment, Tv-1511 mycelium was heat-shocked at 37℃, 40℃, 43℃, 46℃ and 49℃ for 24 h, followed by inoculation on PDA plates or into PD liquid medium at 28 °C for 72 h. The morphology and size of the Tv-1511 growth circles were analysed. Moreover, Tv-1511 mycelium that had been heat-shocked at 46℃ for 24 h and then inoculated into PD liquid medium at 28℃ for 72 h was collected for transcriptomic analysis. Tv-1511 mycelium cultured at 28℃ was used as the control.

Transcriptomic sequencing was conducted by Novogene Technology Co., Ltd. (Beijing, China). Total RNA was extracted using trizol method and tested for concentration, purity and integrity (quality check). Subsequently, high-quality RNA was collected for cDNA library construction using the NEBNext® Ultra™ RNA Library Prep Kit (NEB, Ipswich, MA, USA). The cDNA library was then sequenced using an Illumina Hiseq platform (Illumina, San Diego, CA, USA). The quality of these raw data was evaluated, and the retained clean reads were aligned to the *T*. *viride* Tv-1511 reference genome sequence (GenBank Accession No. VCEC00000000; BioProject: PRJNA543939; BioSample: SAMN11791795) using TopHat (Kim et al. 2013). Data analysis followed the methods described in our previous study (Huang et al. 2022). We conducted differential gene expression (DGE) analysis using DESeq R (1.18.0), Gene Ontology (GO) functional enrichment using Goseq R package software and Kyoto Encyclopedia of Genes and Genomes (KEGG) pathway analyses using KOBAS tool. The Benjamini and Hochberg algorithm was used to correct the *P*-values, with adjusted *P*-values below 0.05 considered statistically significant.

### Semi-quantitative RT-PCR and quantitative real-time RT-PCR assays

Tv-1511 mycelium was collected for RNA extraction. Total RNA was reverse-transcribed to obtain cDNA, which was subsequently used for semi-quantitative RT-PCR and quantitative real-time RT-PCR (qRT-PCR) assays, following the method described by Huang et al. (2018). For semi-quantitative RT-PCR, the optimal annealing temperature and cycle number were 60℃ and 26, respectively. For qRT-PCR, the relative expression levels of genes were analysed using the 2^−ΔΔCt^ method (Livak et al. 2001), with the *T*. *viride β-Actin* gene serving as the internal reference gene. Specific primers were designed based on transcriptome sequencing results, and their sequences are listed in Supplemental Table S1 ​.

### Generation and identification of transgenic *T*. *viride* Tv-1511 over-expressing *TvHSP70*

The open reading frame of *TvHSP70* was amplified using specific primers (Supplemental Table S1) and inserted into the expression vector pBARGPE1-Hygro through the *BamH*I and *EcoR*I sites. The resulting recombinant plasmid, pBARGPE1-Hygro-TvHSP70, was then transformed into protoplasts of *T*. *viride* Tv-1511 using polyethylene glycol (PEG)-mediated transformation, following the method described by Li et al. (2022). The wild-type (WT) strains of Tv-1511 served as controls. The integration and expression of the transgene into the Tv-1511 genome were confirmed through anti-hygromycin screening and qRT-PCR analysis. For anti-hygromycin screening, putative transformants were inoculated on PDA plates containing hygromycin B (300 µg/mL) for three consecutive generations to obtain stable transformants. The selected positive transformants were isolated using a single spore method and cultured in PDA medium for molecular and morphological identification. The *TvHSP70* transcript expression level was detected using the qRT-PCR method described above. Mycelia morphology was observed with an optical microscope.

### Analysis of heat resistance of TvHSP70-OE engineered strains

TvHSP70 over-expressing (TvHSP70‑OE) mutant strains (OE-5, OE-7 and OE-11) and original wild-type (WT) strains of *T. viride* Tv‑1511 were subjected to morphological and heat resistance analyses. Mycelia from WT and transgenic strains (OE-5, OE-7 and OE-11) were inoculated on PDA plates at 28 °C for 60 h to identify their morphological characteristics. Moreover, mycelia from both strains were inoculated on PDA plates or into a PD liquid medium for the heat stress test, following the method described above. The colony diameter, fresh weight (FW) and dry weight (DW) of Tv‑1511 were recorded, and mycelia morphology was observed.

### Analysis of anti-fungal activity of TvHSP70-OE engineered strains

The anti-fungal activity of WT and transgenic strains (TvHSP70-OE) of *T*. *viride* Tv‑1511 on four pathogenic fungi (*Fusarium oxysporum*, *Fusarium moniliforme*, *Botryosphaeria dothidea* and *Botrytis cinerea*) were evaluated using a dual-culture method. Pathogenic fungi and Tv‑1511 (WT, TvHSP70-OE) were inoculated symmetrically on both sides of a petri dish, and the plates inoculated with pathogenic fungi alone served as controls. The colony diameter was measured, and the inhibitory rate was calculated. Fifteen genes related to antibiotic synthesis were selected for gene expression analyses. *T*. *viride β*-*actin* served as the internal control. All primers used are listed in Supplemental Table S1.

### Germination and seedling growth tests

Seed germination and hydroponic seedling tests were conducted to investigate the effect of the TvHSP70-OE engineered strain on seed germination and seedling growth in cucumbers. Healthy and plump cucumber seeds were disinfected with 75% alcohol for 30 s, treated with 2% NaClO for 3 min, and washed with sterile water 4–5 times. Subsequently, the sterilised seeds were germinated on sterilised filter paper with a 5 mL test solution, which contained a 10^5^ CFU (colony forming units)/mL Tv‑1511 (WT or TvHSP70-OE) spore solution. Sterile water served as the control. The radicle length was measured, and germinated seeds were counted. Then, seedlings with uniform growth were selected for the hydroponic seedling test. The seedlings were watered with a 1/2× Hoagland nutrient solution supplemented with the 10^5^ CFU/mL Tv‑1511 (WT or TvHSP70-OE) spore solution. Root length, leaf length, leaf width and stem diameter were recorded.

### Statistical analyses

All statistical analyses were performed using SPSS software (Version 20.0; IBM Corporation, Armonk, NY, USA). Statistical significance was determined using independent samples *t*-test and one-way ANOVA. A *P*-value less than 0.05 was considered statistically significant.

## Results

### Effect of heat stress on *T*. *viride* Tv-1511

**Fig. 1:**
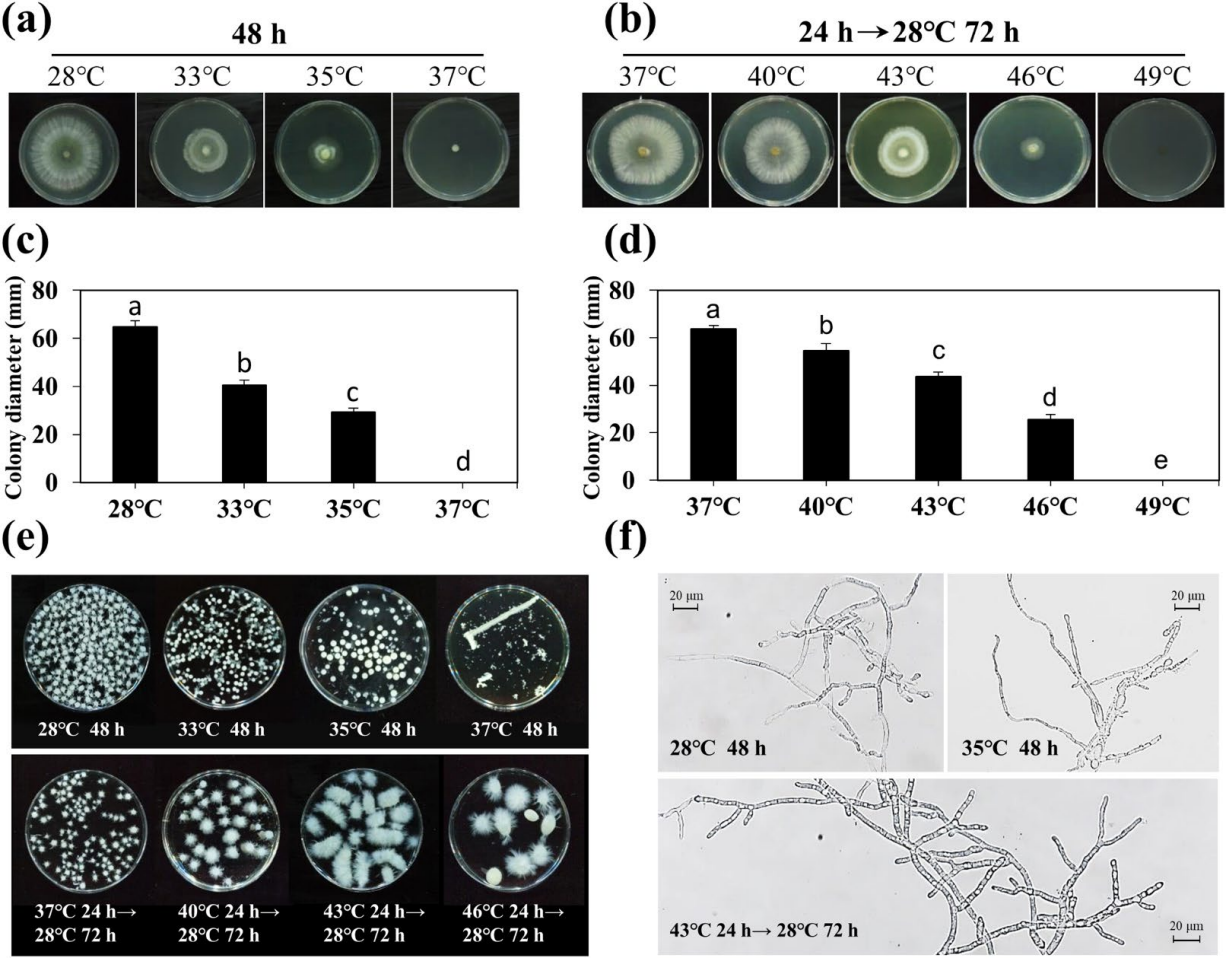
The growth and morphology of *T. viride* Tv-1511 under various heat treatments. **(a)** Plate growth and **(c)** colony diameter of Tv-1511 under constant heat stress (33℃, 35℃ and 37℃). **(b)** Plate growth and **(d)** colony diameter of Tv-1511 under short-term exposure to heat stress (37℃, 40℃, 43℃, 46℃ and 49℃). **(e)** The spherical shape and **(f)** mycelia morphology of Tv-1511 mycelium under various heat treatments; scale bar = 20 μm. The data shows the mean ± S.E. of triplicate experiments. Columns with different letters indicate significant differences at *P* < 0.05 (Duncan’s test)

Our preliminary experiments involving different temperatures suggested that the optimal growth temperature for Tv-1511 was 28℃. Any increase in temperature significantly affected mycelial growth. We observed almost 50% inhibition in the growth of Tv-1511 after 48 h of treatment at 35℃ and hardly any growth at 37℃ (Fig. 1a and c). The spherical shape of Tv-1511 mycelia increased and smoothened as the temperature increased,but the quantity decreased (Fig. 1e). When Tv-1511 was cultured in a liquid medium at 35℃ for 48 h, the mycelium became thinner and produced fewer branches (Fig. 1f). The effect of short-term exposure to heat stress (37℃, 40℃, 43℃, 46℃ and 49℃) on Tv-1511 growth was also investigated. Compared with Tv-1511 subjected to heat shock at 37℃ for 24 h, the inhibition rate reached almost 60% after treatment at 46℃ for 24 h (Fig. 1b and d). The spherical shape of the Tv-1511 mycelia became larger and irregular with increasing temperature (Fig. 1e). More septal branches and thicker mycelia were observed in Tv-1511 when subjected to short-term heat stress (Fig. 1f). Therefore, a high-temperature environment caused significant changes in the growth and morphology of *T. viride* Tv-1511.

### Transcriptomic analysis of *T*. *viride* Tv-1511 under heat stress

**Fig. 2:**
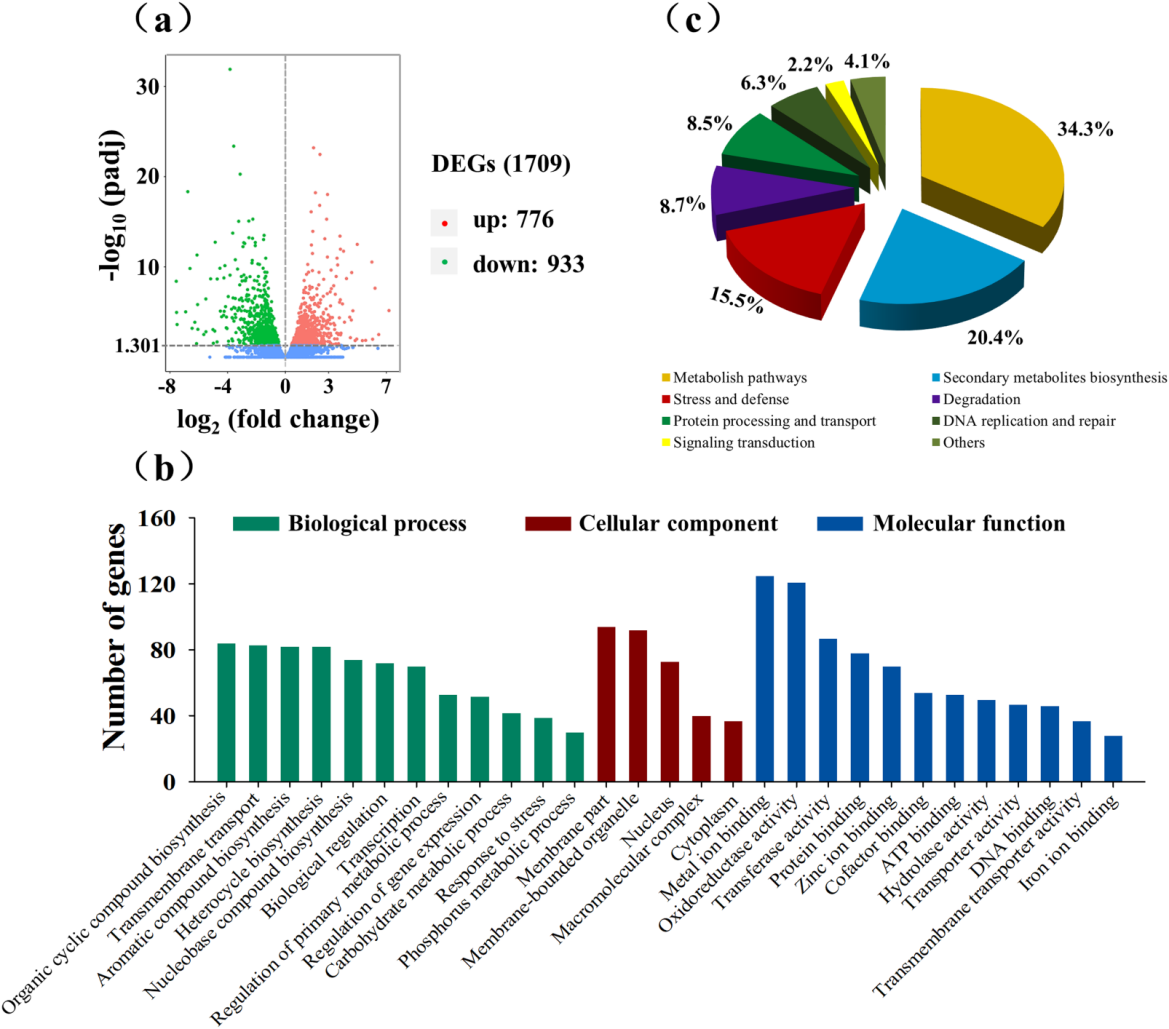
Transcriptomic analysis of *T. viride* Tv-1511 under heat stress. **(a)** Volcano plot of DEGs. **(b)** GO classifications of DEGs. **(c)** Functional classifications of DEGs. DEG: differentially expressed gene; GO: gene ontology

**Fig. 3:**
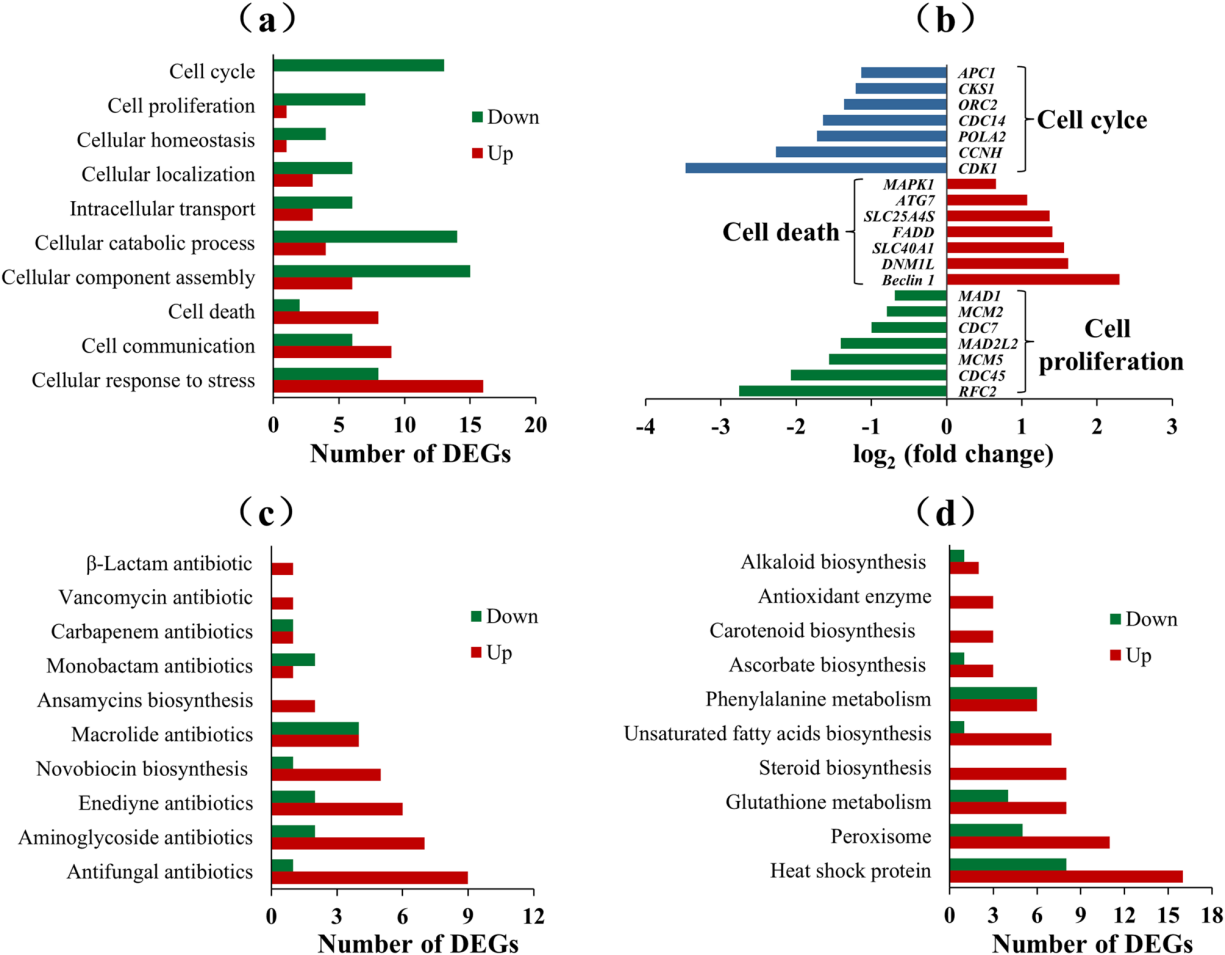
Classifications and expression profiles of differentially expressed genes (DEGs) involved in the main metabolic pathways. **(a)** Classifications of DEGs related to cell growth and development. **(b)** Expression profiles of DEGs associated with the cell cycle, proliferation and death. Classifications and expression profiles of DEGs related to **(c)** antibiotic biosynthesis and **(d)** the anti-oxidant system

Transcriptomic analysis was conducted to identify gene changes in *T. viride* Tv-1511 under heat stress. We identified 1709 differentially expressed genes (DEGs), comprising 776 up-regulated genes and 933 down-regulated genes (Fig. 2a). The transcriptomic results as revealed by GO enrichment and KEGG analyses, showed that these DEGs were mainly related to ‘stress and defence’, ‘secondary metabolite biosynthesis’ and ‘cell growth and development’ (Fig. 2b and c). In the ‘cell growth and development’ category, genes associated with the cell cycle and cell proliferation were down-regulated, whereas genes related to cell death were up-regulated. In the ‘cellular response to stressstress’ group, 16 genes were up-regulated, and 8 were down-regulated (Fig. 3a and b). The ‘secondary metabolite biosynthesis’ pathway accounted for 20.4% of the identified DEGs, including 50 DEGs related to antibiotic biosynthesis. Among these antibiotics, anti-fungal antibiotics were the most abundant, followed by aminoglycoside antibiotics and enediyne antibiotics (Fig. 3c). Some DEGs related to non-enzymatic anti-oxidant systems were also identified. Six steroid biosynthesis genes (*ERG4*, *HSD17*, *ERG5*, *NSDHL*, *SOAT* and *SC5DL*), six unsaturated fatty acid biosynthesis genes (*ELO3*, *PHS1*, *TER*, *SCD*, *fasA* and *fasB*), four glutathione metabolism genes (glutathione synthase [*GSS*], glutathione S-transferase [*GST*], glutathione reductase [*GSR*] and *CHAC*), three ascorbate biosynthesis genes (*ALDH*, myo-inositol oxygenase [*MIOX*] and *UGDH*), three carotenoid biosynthesis genes (*CCD*, *carD* and *CAO2*) were significantly up-regulated (Table 1). Moreover, 24 heat shock protein-encoding genes, 16 peroxisome-related genes and three anti-oxidant enzyme genes were significantly changed in Tv-1511 (Fig. 3d).

**Table 1:** DEGs involved in non-enzymatic capacity ROS-scavenging mechanisms

Gene name	Protein name	Gene ID	log_2_FC	*p*-value
*Glutathione metabolism*				
GSS	Glutathione synthase	A2288	2.23	1.65E-03
GST	Glutathione S-transferase	A0315	2.78	1.78E-03
GSR	Glutathione reductase	A5843	2.64	2.41E-05
CHAC	γ-Glutamyl cyclotransferase	A2207	1.40	1.91E-03
FGH	*S*-Formylglutathione hydrolase	A1998	-1.97	2.13E-04
HAGH	Hydroxyacylglutathione hydrolase	A0785	-1.73	2.52E-03
*Ascorbate biosynthesis*				
ALDH	Aldehyde dehydrogenase	A5010	2.14	3.13E-04
MIOX	Myo-inositol oxygenase	A6117	3.05	5.88E-04
UGDH	UDPglucose 6-dehydrogenase	A1501	1.90	1.21E-03
LGD1	L-Galactonate dehydratase	A0149	-1.13	1.52E-04
*Carotenoid biosynthesis*				
CCD	Carotenoid cleavage dioxygenase	A4367	2.41	1.02E-03
carD	Beta-apo-4’-carotenal oxygenase	A0910	2.30	6.88E-03
CAO2	Torulene dioxygenase	A2948	2.05	1.14E-06
*Alkaloid biosynthesis*				
SRM	Spermidine synthase	A2397	2.52	5.12E-03
AOC2	Primary-amine oxidase	A0948	1.50	6.21E-03
*Phenylalanine metabolism*				
pheA	Prephenate dehydratase	A0027	2.41	2.89E-05
aroG	3-Deoxy-7-phosphoheptulonate synthase	A5867	1.97	1.29E-05
PheRS	Phenylalanyl-tRNA synthetase	A2813	1.75	4.17E-04
COMT	Catechol O-methyltransferase	A2447	-1.18	1.27E-06
*Steroid biosynthesis*				
ERG4	Delta-24-sterol reductase	A3367	2.43	6.07E-03
HSD17	17 β-Hydroxysteroid dehydrogenase	A6793	1.90	1.38E-03
ERG5	Sterol 22-desaturase	A3823	1.87	3.54E-03
NSDHL	Sterol-4α-carboxylate 3-dehydrogenase	A3438	1.57	1.59E-03
SOAT	Sterol-*O*-acyltransferase	A1327	1.27	1.77E-04
SC5DL	Delta7 sterol C-5 desaturase	A6631	0.92	3.17E-03
*Unsaturated fatty acids biosynthesis*				
ELO3	Fatty acid elongase 3	A0862	2.84	6.76E-03
PHS1	3-Hydroxyacyl-CoA dehydratases	A2228	2.12	3.99E-04
TER	Very-long-chain enoyl-CoA reductase	A2243	1.86	3.54E-03
SCD	Stearoyl-CoA desaturase	A3841	1.51	1.59E-03
fasA	Fatty acid synthase subunit alpha	A4278	1.34	1.77E-04
fasB	Fatty acid synthase subunit beta	A4289	1.23	3.17E-03

We identified and classified transcription factors (TFs) involved in the heat stress response to Tv-1511, identifying 437 TFs belonging to 63 TF families. Among these families, MFS_1 was the most abundant, followed by Fungal_trans and p450. In the HSP70 and AMP-binding families, up-regulated genes significantly outnumbered the down-regulated genes (Supplemental Fig. S1). These results indicate that heat stress inhibits Tv-1511 growth by decreasing the cell number and size, resulting from the down-regulation of genes involved in cell cycle and cell proliferation. Simultaneously, heat stress can trigger various stress responses, including the anti-oxidant defence system, HSPs, stress-response-related TFs and significantly activated antibiotic biosynthesis.

### Analysis of key genes involved in heat stress response in *T*. *viride* Tv-1511

**Fig. 4:**
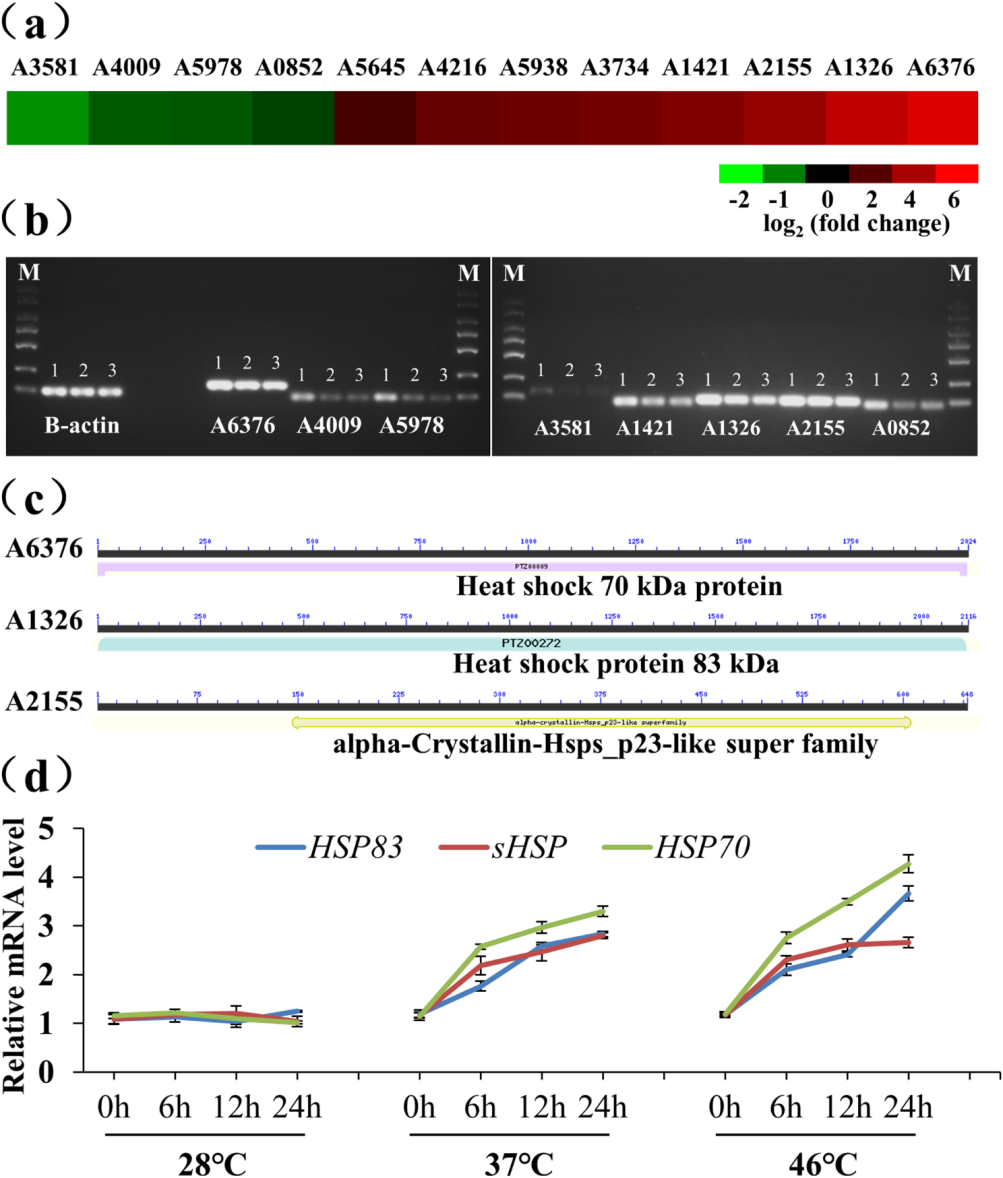
Expression analysis of heat shock protein (HSP) genes in *T. viride* Tv-1511 under heat stress. **(a)** Expression profiles of differentially expressed genes (DEGs) related to HSP genes. **(b)** RT-PCR analysis of HSP genes under different temperature treatments. **(c)** Conserved structural domain analysis of the three HSPs. **(d)** Expression levels analysis of *HSP70*, *HSP83* and *sHSPs* under different temperature treatments. M: DL2000 marker; 1, 2 and 3 indicate different temperature treatments (28 °C, 37 and 46 °C, respectively)

Further analysis was conducted to evaluate HSP gene expression. We identified 12 DEGs, comprising 8 up-regulated and 4 down-regulated genes (Fig. 4a). Among these up-regulated genes, three genes (A2155, A1326 and A6376) maintained high expression levels under high-temperature treatment, consistent with the transcriptomic results (Fig. 4b). All three protein sequences contained conserved structural domains and shared a high degree of homology with HSPs. Finally, A6376 was identified as *HSP70*, A1326 *HSP83* and A2155 s*HSPs* (Fig. 4c). To verify the expression levels of the three genes under high-temperature treatment, we conducted the qRT-PCR analysis. *HSP70*, with a high expression level, was identified as a key gene in Tv-1511 that responded to heat stress (Fig. 4d). We isolated *HSP70* from *T*. *viride* Tv-1511, designating it as *TvHSP70*.

### Generation of TvHSP70-OE engineered strain of *T*. *viride* Tv-1511

**Fig. 5:**
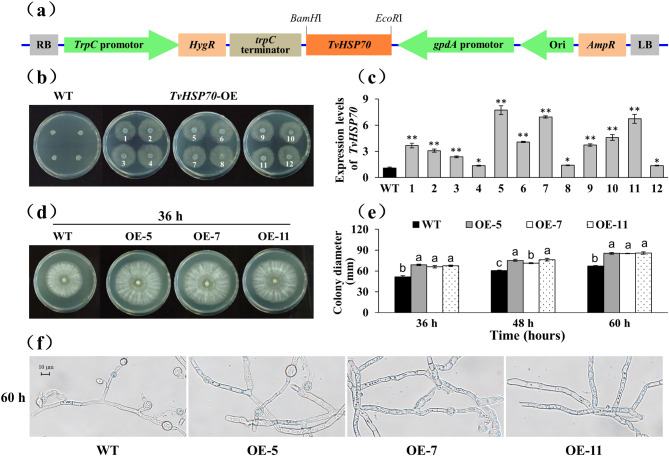
Generation and identification of transgenic *T. viride* Tv-1511 over-expressing *TvHSP70*. **(a)** Diagram depicting the over-expression vectors. **(b)** Anti-hygromycin screening. **(c)** Relative expression of *TvHSP70*. **(d)** Plate growth, **(e)** colony diameter and **(f)** mycelia morphology of transgenic strains compared with WT strains; scale bar = 10 μm. WT: wild type; TvHSP70‑OE: *TvHSP70* over-expressing; OE-5, OE-7 and OE-11: transgenic strains with over-expressed *TvHSP70*; Data are presented as mean ± S.E. of triplicate experiments; Columns with different letters indicate significant differences at *P* < 0.05 (Duncan’s test); * and ** indicate a significant difference from that of WT at *P* < 0.05 and *P* < 0.01, respectively

TvHSP70-OE engineered strains were generated by transforming the recombinant vector pBARGPE1-Hygro-*TvHSP70* through PEG-mediated protoplast transformation. The transgenes were constitutively expressed under the control of *gpdA* promoter and *trpC* terminator (Fig. 5a). Putative transformants were selected in the presence of 300 µg/mL hygromycin B for three consecutive generations, yielding 12 positive transformants (Fig. 5b). Compared with the WT strains, the *TvHSP70* expression level was significantly increased in these positive transformants. Among the transgenic strains, OE-5 exhibited the highest expression, followed by OE-7 and OE-11 (Fig. 5c). The transgenic strains showed noticeable growth advantages compared with the WT strains (Fig. 5d and f). The growth diameters of the WT strains averaged 51.39 cm, whereas those of the transgenic strains displayed a 1.34-, 1.26- and 1.31-fold increase in OE-5, OE-7 and OE-11, respectively (Fig. 5e). The transgenic strains formed thicker and more compact hyphae (Fig. 5f). These results indicate that *TvHSP70* significantly promotes *T*. *viride* Tv-1511 growth.

### Effect of over-expressing *TvHSP70* on heat resistance in *T*. *viride* Tv-1511

**Fig. 6:**
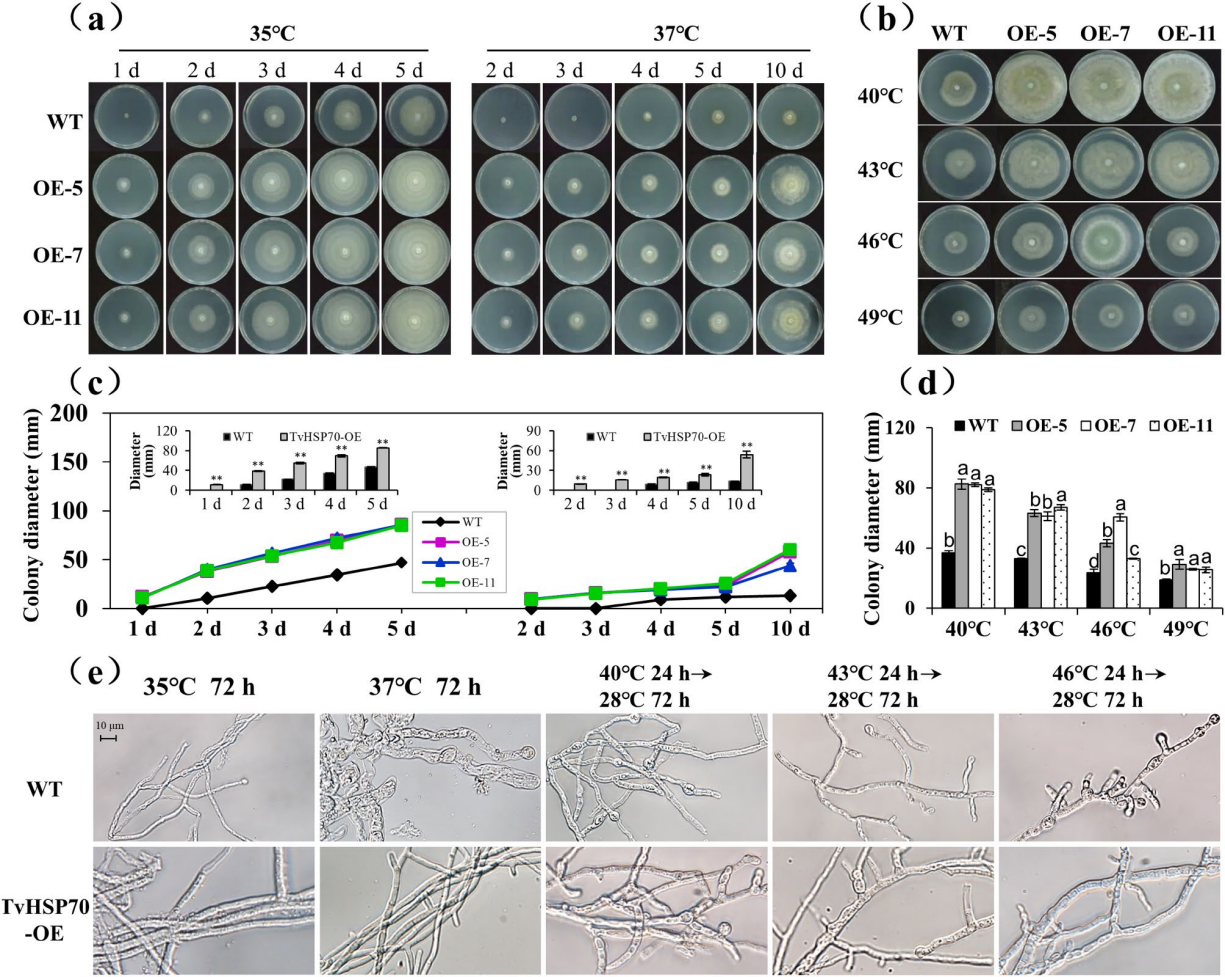
Analysis of heat resistance in TvHSP70-OE engineered strains and WT strains under various heat treatments. **(a)** Plate growth and **(c)** colony diameter of transgenic and WT strains under constant heat stress (35℃ and 37℃). **(b)** Plate growth and **(d)** colony diameter of transgenic and WT strains under short-term exposure to heat stress (37℃, 40℃, 43℃, 46℃ and 49℃). **(e)** Mycelia morphology of transgenic and WT strains under various heat treatments; scale bar = 10 μm. WT: wild type; TvHSP70‑OE: TvHSP70 overexpressing; OE-5, OE-7 and OE-11: transgenic strains with over-expressed *TvHSP70*; Data are presented as mean ± S.E. of triplicate experiments; Columns with different letters indicate significant differences at *P* < 0.05 (Duncan’s test); * and ** indicate a significant difference from that of WT at *P* < 0.05 and < 0.01, respectively

To illustrate the role of *TvHSP70* in the adaptation of Tv-1511 to heat stress, we performed heat resistance analysis of transgenic and WT strains. In the constant heat stress assay, the growth of WT strains was severely inhibited at 35 °C and almost lost its viability at 37℃. In contrast, the TvHSP70-OE engineered strains maintained relatively normal growth, even after 10 d of treatment (Fig. 6a). Compared with WT, the colony diameter increased 1.82- and 4.09-fold in transgenic strains after treatment at 35℃ for 5 d and 37℃ for 10 d (Fig. 6c). Similar patterns were also observed in short-term acute heat stress assays. Severe growth inhibition was observed in WT strains under short exposure to heat stress (40℃, 43℃, 46℃ and 49℃ for 24 h), whereas these symptoms were effectively alleviated in TvHSP70-OE engineered strains (Fig. 6b). Transgenic strains exhibited 2.21-, 1.93-, 1.94- and 1.42-fold increases compared with WT strains under treatments at 40℃, 43℃, 46℃ and 49℃, respectively (Fig. 6d). The heat stress-induced damage to mycelia morphology was visualised using an optical microscope. Interestingly, under the same conditions, the transgenic strains showed significant advantages regarding hyphae numbers and septal plates compared with the WT strains (Fig. 6e). Based on FW and DW measurements, the transgenic strains showed significantly higher biomass than the WT strains (*P* < 0.05) (Supplemental Fig. S2). These results imply that *TvHSP70* over-expression triggered a significant increase in *T. viride* Tv-1511 growth and biomass under heat stress.

### Effect of over-expressing *TvHSP70* on the anti-oxidant defence system of *T*.*viride* Tv-1511

**Fig. 7:**
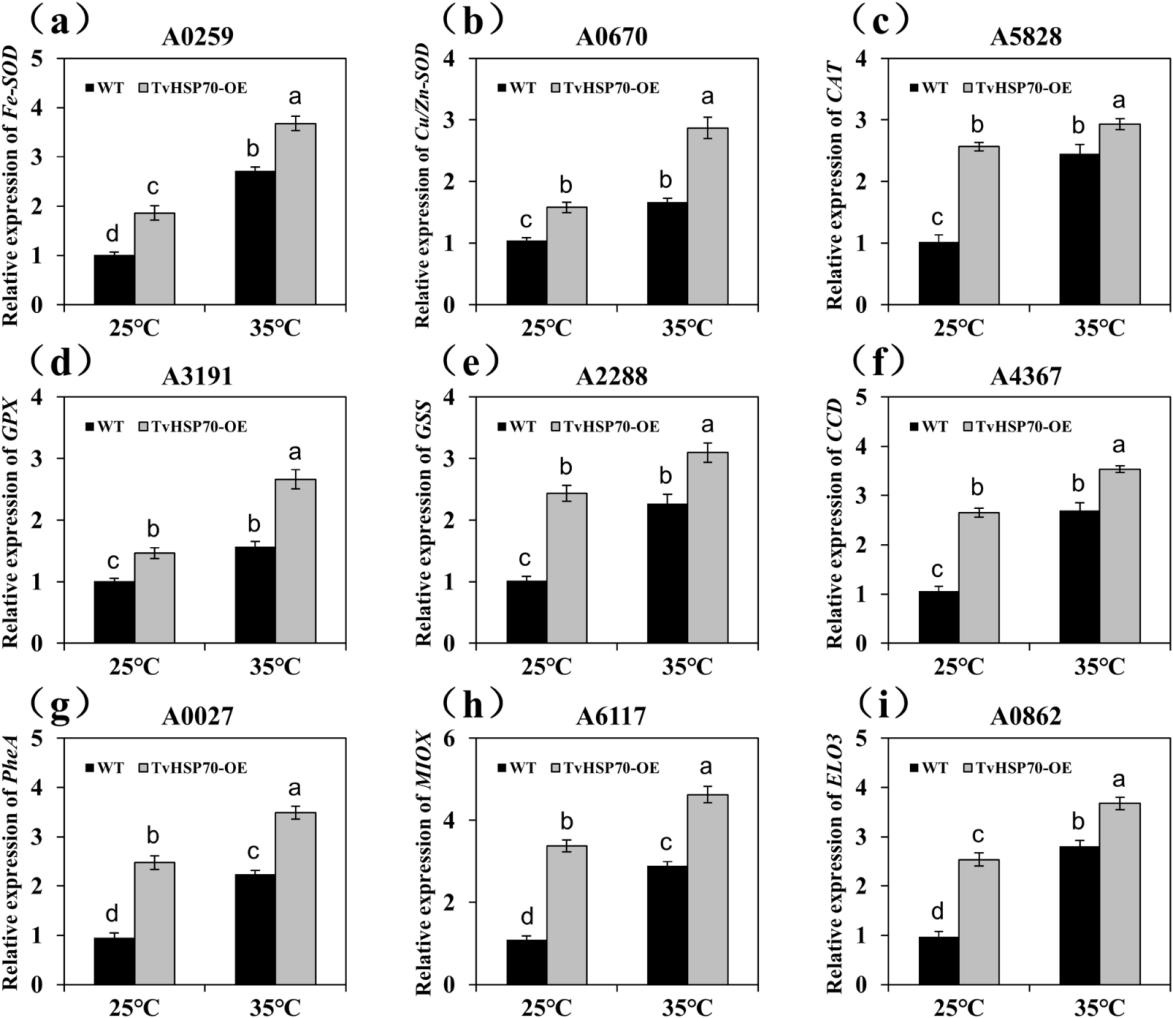
Expression level analysis of genes involved in the anti-oxidant defence system in TvHSP70-OE engineered and WT strains under heat stress treatments. Expression levels of **(a)***Fe-SOD*, **(b)***Cu/Zn-SOD*, **(c)***CAT*, **(d)***GPX*, **(e)***GSS*, **(f)***CCD*, **(g)***PheA*, **(h)***MIOX* and **(i)***ELO3*. WT: wild type; TvHSP70‑OE: transgenic strains with *TvHSP70* overexpressed; Data are presented as mean ± S.E. of triplicate experiments; Columns with different letters indicate significant differences at *P* < 0.05 (Duncan’s test)

After identifying genes involved in the anti-oxidant system, we analysed their transcript levels using qRT-PCR for the transgenic and WT strains. Four anti-oxidant enzyme genes (Fe-superoxide dismutase [*Fe-SOD*], CuZn-superoxide dismutase [*Cu/Zn-SOD*], catalase [*CAT*] and glutathione peroxidase [*GPX*]) were significantly up-regulated (*P* < 0.01) in the transgenic strains, with an approximately 1.66-fold increase in expression (Fig. 7a and d). *TvHSP70* over-expression also potentially affected the non-enzymatic defence systems in *T. viride* Tv-1511. We found that several genes involved in the biosynthesis of unsaturated fatty acids were significantly activated in transgenic strains under normal and heat stress conditions. These genes encompassed the *GSS* gene responsible for glutathione synthesis, the carotenoid cleavage dioxygenase 4 gene functioning in carotenoid biosynthesis, the prephenate dehydratase (*PheA*) gene associated with phenylalanine metabolism, the *MIOX* gene related to ascorbate biosynthesis and the fatty acid elongase 3 (*ELO3*) gene (Fig. 7e and i). These results further confirmed that the over-experssion of *TvHSP70* triggered enzymatic and non-enzymatic anti-oxidant defence mechanisms in *T. viride* Tv-1511 to cope with heat stress.

### Effect of over-expressing *TvHSP70* on anti-fungal activity of *T*. *viride* Tv-1511

**Fig. 8:**
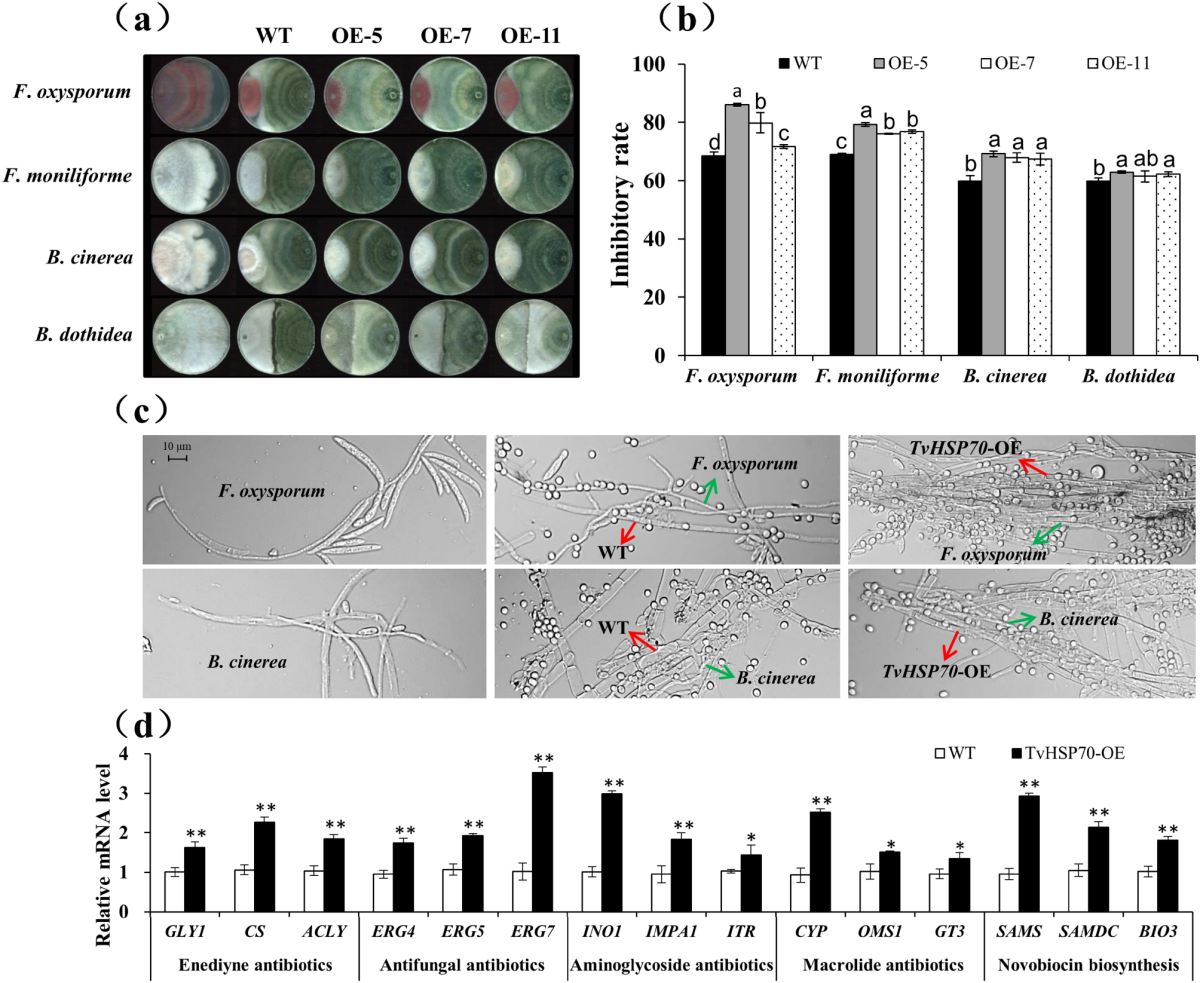
Anti-fungal activity analysis of TvHSP70-OE engineered and WT strains. **(a)** Plate confrontation and **(b)** inhibitory rates of transgenic and WT strains against four pathogenic fungi. **(c)** Microscopic observation of the mycelia morphology of transgenic and WT strains against two pathogenic fungi; scale bar = 10 μm. **(d)** Expression levels analysis of genes related to antibiotic synthesis. WT: wild type; TvHSP70‑OE: TvHSP70 overexpressing; OE-5, OE-7 and OE-11: transgenic strains with over-expressed *TvHSP70*; Data are presented as mean ± S.E. of triplicate experiments; * and ** indicate a significant difference from that of WT at *P* < 0.05 and < 0.01

We previously discovered that heat stress induced antibiotic biosynthesis. To illustrate the effect of *TvHSP70* on the ant-ifungal activity of Tv-1511, we evaluated the antagonistic activities of the WT and transgenic strains. Notably, TvHSP70-OE engineered strains exhibited more effectiveness than WT strains against the growth of four pathogenic fungi (Fig. 8a). The inhibitory rates of the transgenic strains in *F*. *oxysporum*, *F*. *moniliforme*, *B*. *cinerea* and *B*. *dothidea* averaged 79.2%, 77.4%, 68.1% and 62.2%, respectively, which were 1.16, 1.12, 1.14 and 1.04 times higher than those of the WT strains (Fig. 8b). Moreover, mycelia morphology observed with an optical microscope suggested that the antagonistic mechanisms of the WT and transgenic strains of *T*. *viride* Tv‑1511 are competition, parasitism and antibiosis (Fig. 8c).

To reveal the molecular mechanisms underlying these antagonistic activities, we screened 15 genes related to antibiotic synthesis through transcriptomic analysis and conducted gene expression analyses. Three genes involved in the biosynthesis of enediyne antibiotics (*GLY1*, *CS* and *ACLY*), anti-fungal antibiotics (*ERG4*, *ERG5* and *ERG7*), aminoglycoside antibiotics (*INO1*, *IMPA1*and *ITR*), macrolide antibiotics (*CYP*, *OMS1* and *GT3*), and novobiocin antibiotics (*SAMS*, *SAMDC* and *BIO3*) were significantly up-regulated (*P* < 0.01 and *P* < 0.05) in the transgenic strains (Fig. 8d). In particular, the expression levels of *ERG7*, *INO1* and *SAMS* in the transgenic strains were 3.52, 2.98 and 2.93 times higher than those in the WT strains, respectively. Therefore, TvHSP70 played crucial roles in regulating the anti-fungal activity of *T. viride* Tv‑1511 by up-regulating genes involved in the biosynthesis of enediyne, anti-fungal, aminoglycoside, macrolide and novobiocin antibiotics.

### Effect of TvHSP70-OE engineered strains on seed germination and seedling growth in cucumber under heat stress

**Fig. 9:**
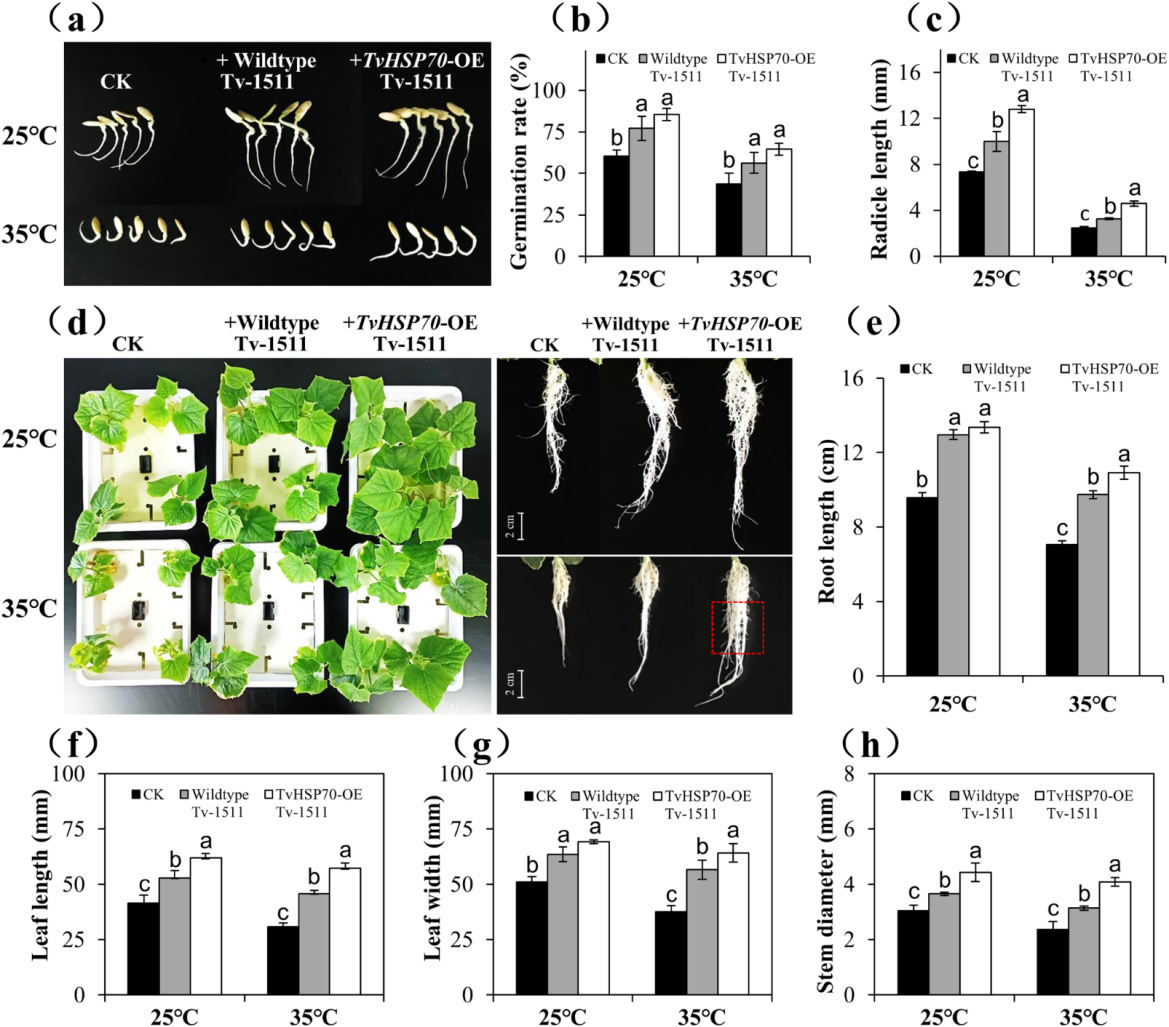
Analysis of the plant-growth-promoting ability of TvHSP70-OE engineered and WT strains under heat stress and normal conditions. **(a)** Germination phenotype, **(b)** germination rate and **(c)** radicle length of cucumber seeds. **(d)** Changes in the phenotypic characters of the shoots and roots of cucumber seedlings. **(e)** Root length, **(f)** leaf length, **(g)** leaf width and **(h)** stem diameter of cucumber seedlings. CK: cucumber treated with sterile water. + Wild-type Tv-1511: cucumber treated with the original WT strain of *T. viride* Tv‑1511. + TvHSP70-OE Tv-1511: cucumber treated with TvHSP70-OE engineered strains of *T. viride* Tv‑1511. Data are presented as mean ± S.E. of triplicate experiments; Columns with different letters indicate significant differences at *P* < 0.05 (Duncan’s test)

In the seed germination assay, we found that cucumber treated with WT and TvHSP70-OE engineered strains exhibited an average germination rate under heat stress (35℃) and normal (25℃) conditions (Fig. 9b). However, cucumber treated with TvHSP70-OE engineered strains showed notable increases in radicle length (Fig. 9a). Comparatively, under heat stress and normal conditions, the radicle lengths of cucumbers treated with WT strains were 1.36 and 1.33 times that of cucumbers treated with sterile water (CK). In contrast, the radicle lengths of the cucumbers treated with transgenic strains were 1.74 and 1.86 times higher than those of CK (Fig. 9c). The damage induced by high temperatures was visualised by the growth state of the roots and leaves. The control plants exhibited wilting and almost dead roots, whereas these symptoms were effectively alleviated in cucumbers treated with WT and TvHSP70-OE engineered strains (Fig. 9d). Additionally, based on the growth parameters, cucumbers treated with TvHSP70-OE engineered strains showed noticeable growth advantages under heat stress (Fig. 9e and h). These results confirmed that over-expression of *TvHSP70* improved the growth-promoting ability of *T. viride* under heat stress.

## Discussion

The filamentous fungi genus *Trichoderma* spp., found in different ecological niches with multiple capabilities, face constant threats from various abiotic stresses in their industrial and agricultural applications, particularly heat stress. Most *Trichoderma* species are mesophilic, and high temperatures significantly affect mycelial growth and spore germination (Singh et al. 2014). Consistent with this observation, we observed that *T*. *viride* Tv-1511 behaves as a mesophilic fungus with an optimal growth temperature of 28℃. Further temperature increases caused significant changes in the growth and morphology of Tv-1511. Exploring the molecular basis for the impact of heat stress on Tv-1511 growth is of significant interest. In this study, all identified DEGs related to cell growth and development were categorised into ten functional groups. Notably, all DEGs involved in the cell cycle and most DEGs involved in cell proliferation were down-regulated. Conversely, most DEGs associated with cell death were up-regulated. The expression patterns of seven cell cycle regulatory genes (Xiao et al. 2021; Riba et al. 2022), seven cell-death-associated genes (Khafif 2014; Rantong and Gunawardena. 2015) and seven cell proliferation-related genes (Mandal and Chaurasia. 2021; Ma et al. 2023) were consistent with the decrease observed in the growth rate of Tv-1511 under heat stress. These results indicated that heat stress significantly affected the mycelial growth of Tv-1511 by decreasing cell number and size as a result of the down-regulation of genes involved in cell proliferation and the cell cycle, as well as the up-regulation of genes involved in cell death. Additionally, heat stress affects the defence systems of Tv-1511. Numerous DEGs were enriched in the ‘stress and defence’ category, accounting for 15.5% of all identified DEGs, and most of these genes were up-regulated. Notably, the expression levels of three anti-oxidant enzyme genes, 11 peroxisome-associated genes and 16 HSP-encoding genes were significantly activated in Tv-1511. DEGs related to the biosynthesis of steroids, unsaturated fatty acids, ascorbate, carotenoids and glutathione were significantly up-regulated. Moreover, the general phenylalanine metabolism is crucial in defence responses (Zhan et al. 2022). In this study, we observed numerous DEGs associated with phenylalanine metabolism that showed significant changes. Heat stress also altered the transcription levels of many TFs. Overall, *T*. *viride* Tv-1511 has evolved several defence mechanisms to survive extreme temperature conditions, including anti-oxidant enzymatic properties, non-enzymatic capacity (e.g. steroids, unsaturated fatty acids, ascorbate, carotenoids and glutathione), and the significant activation of stress-response-related genes and TFs.

Among these defence mechanisms, HSPs are pivotal in heat-tolerance mechanisms (Sarkar et al. 2021). HSP70, a ubiquitous and highly conserved protein among various species, plays an important role in the cellular response to heat stress (Płażek et al. 2020). Consistent with this observation, we found that *HSP70* maintains high expression levels and is more sensitive to high-temperature stress than other HSPs. We identified *TvHSP70* as a key gene in *T*. *viride* Tv-1511 that responds to heat stress. The thermal tolerance of organisms has been identified as a complex multi-genic trait that involves multiple physiological and biochemical mechanisms and requires the coordinated and precise activation of numerous genes (Sun et al. 2021; Pudake et al. 2017). However, previous studies on different species, including animals, plants and microorganisms, have shown that over-expressing a single *HSP70* effectively improves thermal stability (D’Souza et al. 2022; Ni et al. 2021; Montero-Barrientos et al. 2010). In our study, transgenic Tv-1511 strains over-expressing *TvHSP70* exhibited significant growth advantages and maintained an improved growth-promoting ability compared with WT strains under heat stress. Moreover, *TvHSP70* over-experssion induced a series of molecular changes that affected the anti-oxidant defence system of *T*. *viride* Tv-1511. The anti-oxidant enzyme-encoding genes (Zainy et al. 2023), *Fe-SOD*, *CuZn*-*SOD*, *CAT* and *GPX* were significantly up-regulated in TvHSP70-OE engineered strains. In addition, several genes involved in glutathione (Yamazaki et al. 2019), carotenoid (Yu and Tian. 2021), ascorbate (Munir et al. 2020), unsaturated fatty acid biosynthesis (Xue et al. 2020) and phenylalanine metabolism (Xu and Zhang. 2020) were significantly activated in the transgenic strains. These results indicated that *TvHSP70* over-experssion triggered enzymatic and non-enzymatic anti-oxidant defence mechanisms in *Tv-1511* to cope with adverse conditions. This result was supported by the findings of previous studies on tobacco (Song et al. 2021)d coli cells (Eunhye et al. 2015), where HSP70 enhanced stress tolerance by improving anti-oxidant ability.

*T*. *viride* is considered the most promising biocontrol agent against various plant pathogens. In this study, the WT and TvHSP70-OE engineered strains of *T*. *viride* Tv-1511 were screened for anti-fungal activity against four selected plant pathogenic fungi (*Fusarium oxysporum*, *F*. *moniliforme*, *B. dothidea* and *B.cinerea*). Consistent with these conclusions, we found Tv-1511 showed significant anti-fungal activity against all tested fungi, with inhibitory rates ranging from 59.7 to 68.9%. The efficiency of Tv-1511 as an antagonist was due to its rapid growth and abundant production of conidia, which significantly inhibited mycelia growth and spore germination in pathogenic fungi. This finding suggests that competition is a potential mechanism of Tv-1511’s antagonistic action against pathogenic fungi, consistent with previous studies (Gusnawaty et al. 2020). The optical microscope showed that *T. viride* Tv-1511 was able to entangle, attach, adsorb and invade the mycelium of the tested fungi, causing mycelial malformation and breakage. This suggested that mycoparasitism may also be involved in the antagonistic mechanisms of *T. viride* Tv-1511. Recent evidence has shown that the *T. viride* strain showed parasitic behaviour against test pathogenic fungi by encircling, shrinking and ultimately degrading the host hyphae (Naglot et al. 2015). In addition, notable inhibition zones and anti-bacterial circles were observed in the confrontation between *T. viride* Tv-1511 and *B. dothidea*, indicating antibiotic production. This finding is consistent with previous studies by Khan et al. (2021) and Gajera et al. (2020), who reported that *T. viride* regulates pathogenic fungi by producing antibiotics and other secondary metabolites. These results indicate that the biocontrol effect of *T. viride* results from various mechanisms, including competition for nutrients and space, direct parasitism and the production of antibiotics.

However, the anti-fungal activity of *Trichoderma* can be strongly influenced by temperature, an a 5 °C increase can significantly impact its biocontrol efficiency (Carro-Huerga et al. 2021). Recent research has shown that temperature can affect the production of secondary metabolites (Lelio et al. 2021), which are considered essential for the anti-fungal activity of *Trichoderma* (Mutawila et al. 2015). Consistent with this conclusion, we found that high temperature induced the biosynthesis of secondary metabolites, particularly antibiotics, in *T*. *viride* Tv-1511. High temperatures enhanced the anti-fungal activity of *Trichoderma* and significantly damaged its growth and survival. To resolve this contradiction, transgenic Tv-1511 strains with high resistance to high-temperature stress were generated, and their anti-fungal activity was evaluated. Interestingly, the transgenic strains showed significant anti-fungal activity and increased expression of genes encoding proteins involved in the biosynthesis of enediyne, anti-fungal and aminoglycoside antibiotics. These results suggest that *TvHSP70* plays an important role in regulating anti-fungal activity by up-regulating genes involved in the biosynthesis of fungal, aminoglycoside and macrolide antibiotics.

In this study, we demonstrated that *T. viride*’s survival under heat stress requires the activation of adequate defence mechanisms (e.g. the biosynthesis of secondary metabolites, anti-oxidant defence systems, HSPs and stress-response-related TFs) to prevent impairment of metabolic functions. Our study also conducted comparative morphological, physiological and molecular analyses to determine the possible mechanisms of TvHSP70 in the growth, heat resistance and anti-fungal activity of *T. viride* Tv-1511. *TvHSP70* plays crucial role in regulating anti-fungal activity by up-regulating genes involved in the biosynthesis of fungal, aminoglycoside and macrolide antibiotics biosynthesis. Regarding heat resistance, *TvHSP70* triggered enzymatic (e.g. Fe-SOD, Mn-SOD, CAT and GPX) and non-enzymatic (e.g. glutathione, carotenoid, ascorbate and unsaturated fatty acids) defence systems. These results provide the basis for an improved understanding of the underlying molecular mechanisms of *HSP70*. Additionally, it provides a theoretical basis for the further biotechnological applications of *Trichoderma* inoculums in plants.

### Electronic supplementary material

Below is the link to the electronic supplementary material.

Supplementary Material 1

## Data Availability

All data generated or analyzed during this study are included in this published article (and its supplementary information files). The raw sequencing data were deposited in the NCBI Short Read Archive (SRA) database (http://www.ncbi.nlm.nih.gov/sra/) under the accession number PRJNA1029905.
