# Supplementary material for: Enhanced resistance to heat and fungal infection in transgenic Trichoderma via over-expressing the HSP70 gene
Source: AMB Express. 2024 Apr 10;14:34. doi: 10.1186/s13568-024-01693-5 (PMC11006649; doi:10.1186/s13568-024-01693-5)
Supplement: Supplementary file 1 — Supplementary Material 1 [file 13568_2024_1693_MOESM1_ESM.pdf]

Enhanced resistance to heat and fungal infection in transgenic *Trichoderma* via over-expressing the *HSP70* gene

AMB Express

Yanhua Huang[1], Changfa Liu[1], Xueuxe Huo[1], XianZhi Lai[1], Wentao Zhu[1], Yongren Hao[1], Zehui Zheng[1]*, KaiGuo[1]*

[1] Biology Institute, Qilu University of Technology (Shandong Academy of Sciences), Jinan 250014, China

*Corresponding author:

Zehui Zheng (zhengzh@qlu.edu.cn)

KaiGuo (guokaicc@163.com).

Fig. S1 Distribution and expression profiles of transcription factors families in *T. viride* Tv-1511 under heat stress. (a) Distribution of transcription factors families. (b) Number of up-regulated and down-regulated transcription factors. TFs: transcription factors.

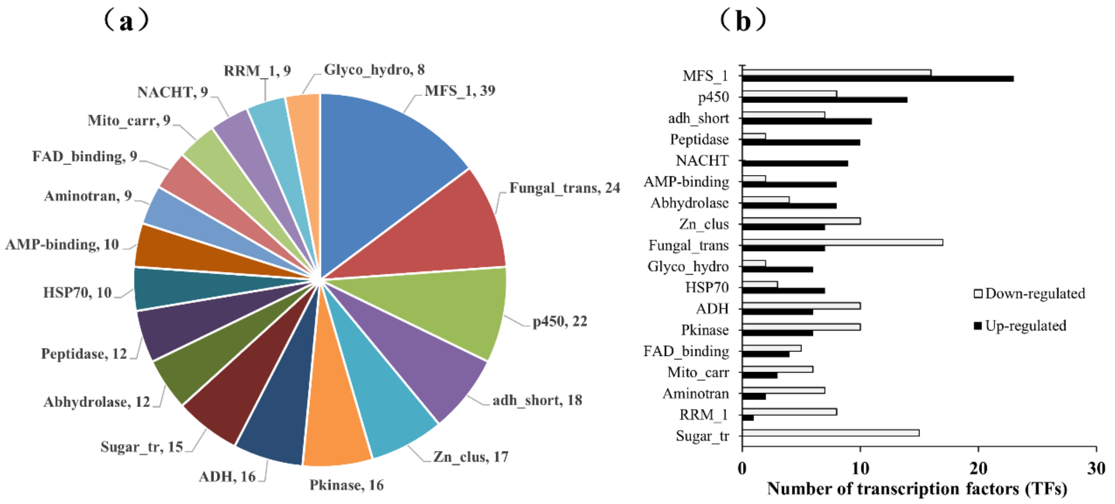

Fig. S2 Effects of heat stress on biomass of TvHSP70-OE engineered and WT strains. WT: wild-type; OE-5, OE-7 and OE-11: transgenic strains with over-expressed *TvHSP70*; Data are presented as mean ±S.E. of triplicate experiments; Columns with different letters indicate significant differences at $P < 0.05$ (Duncan's test).

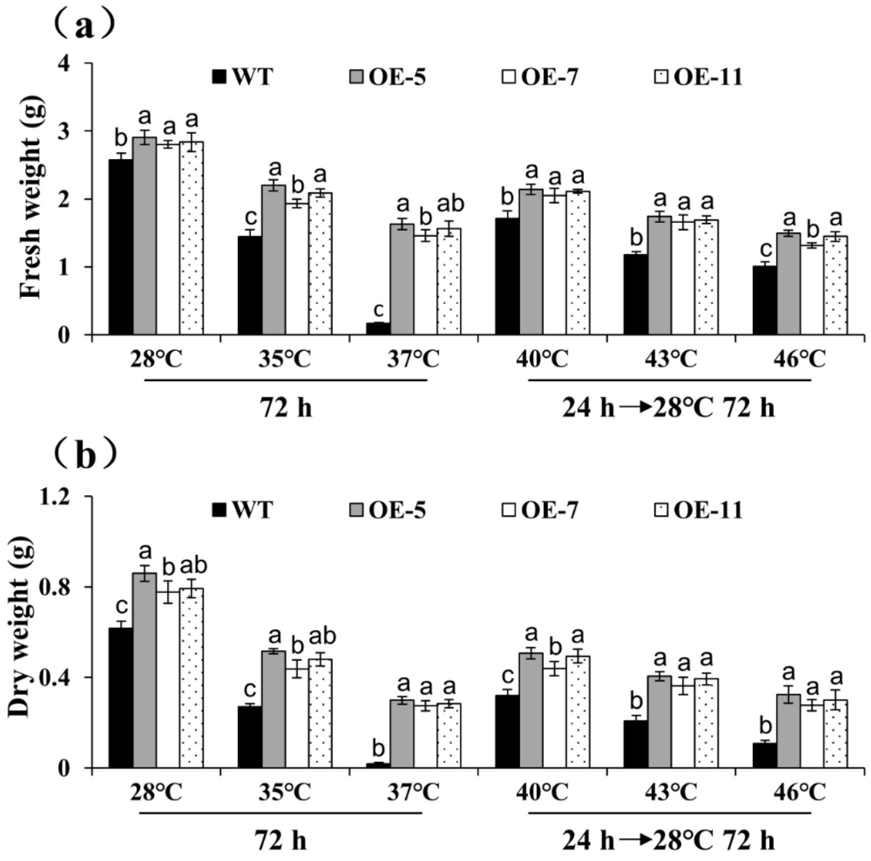

Table S1. Primer sequences used in the experiments

**Gene name**	**Forward primer (5'-3')**	**Forward primer (5'-3')**
β-Actin	TTCGCTCTTCCTCACGC	GATGTCACGGACGATTTC
A6376	TCCGAGGTCCAGGCTGATATGAAG	GTCTCACGCATCTTGGTCAGGATC
A1326	ATCAAGAAGCACTCCGAGTTCATCAG	GGTCTCCTCCTCAGCATCCTCATC
A2155	GCCTAAGAAACAACCTGCCGACTC	CATACCATGACCAGCACCAGGAAC
A4009	GACCAGTCCATCACCATTGCTTCC	TACTTCTCGGAGTCCTCAACCATCTG
A5978	ATTCTCGGCAAGATGAAGGAGGTTG	GTTGAAGTAGGCAGGGACAGTAACG
A3581	ACCTGGAGCACTCACTATACGATACG	TTGGACAATTCCTGGTTGAAGTAGAGC
A1421	ACCAACACCACTATAACAGACACAGAC	GAGAGAGGTCGCAGATGTCGTTTG
A0852	AGCGAGGATTTACAGCGACCAAAG	CTTTTCCCTGAGACCGTGGATGAC
TvHSP70	CGGGATCC ATGTCTATGGGACCAGCCGT	CGGAATTC TTAGTCGACCTCCTCGACGG
GLY1	TCCTGTCGGCAGCATCATTGTTG	GAATGTCTCGTCCACAGCCACTC
CS	AGAAGCACGGCAAGACCAAGAAC	AGGTGGCGGTGTAGTACAGAGTC
ACLY	CCGTGATGGTGACTGGATCTCTTC	GTAGCGGCAATCTCCTCGTTGG
ERG4	CTTCATCGCTTACTTCTCCGCTCTG	AAGGATGCCAAACATACGAGGGTTC
ERG5	TGGCGTGAGGCTGAGGAGAAG	GGTGAAGACAGTCTGGGAGATTTCG
ERG7	GCAAGAGTTGTTTGTTCAGCCTAC	CAGGAGCCAGTTGACCGTGTTG
INO1	ACTGCCAACACTGAGCGATATGC	GCGACAGCAAAGACGGTGGAG
IMPA1	CATGGCTGGCGGCAATCCTG	ACTCCTCCACAAGCTCCGTCTG
ITR	AGAGACTGGATGGCGGTGGATG	TCTGGCATGAAGGCAACAAGAGC
CYP	CGCCGCAGGAGTGGTTCAAG	CGTTGTCCAGGTCATCGGTTGTC
OMS1	CCGTCGCCGTGCTGTCTAAC	GCCATAATAGCCCTTACCGTGTTCC
GT3	ACGCCTACTCCTCGCCTTCAG	CGCAGCCATTGGTAAGCCTCTC
SAMS	GCTCTCCTACGCCATTGGTGTTG	TCGTCAGAGGTCTTGTCAGAGGTG
SAMDC	GTCCGACGCTGTTGATGCCTATC	TCGTGCCGCAAGTCTTCAAGATG
BIO3	GCTCACGGCGATTACTTCCAGAC	TTGAGTCCACCAGGAGGCAGAG
Fe-SOD	AGGGCACCATCACTGACTCTCTG	GCCAGCGTTGCCAGTCTTCAG
Cu/Zn-SOD	CTCCAAGCACCACCAGACGTATG	CGCCGTGGAAGTTGAGCAGAG
CAT	GATGCCCTCGCCCACTTTGAC	ATGGAGGTGAGGTCGGTGATGTC
GPX	ATGTCTTCTGCCACCAACTTCTTG	GTAGGTGAAGCCGCACTTGGAAG
GSS	AACTGATTGTACCGCCTCCTGTTTC	ATCTCTCCGCTGCCCTGGTTC
CCD	GTATGATGCGACGACACCTGAGC	CCTCCTTCCACCTTGCGATTGC
PheA	AACACCACCCGTTTCCTCATTATCG	GGTCCTCGTAGTAGCCTCTCCAAC
MIOX	ATCCGCTACCACTCGTTCTACCC	ACGCTAGGAACATCATCACTCTTGC
ELO3	TCCGAATCTGGTGGAAGGAGTGG	AGTAGGTGTAGGAGGCGAAGTAGAC
